# Driving Distractions Among Public Health Center Clients: A Look at Local Patterns During the Infancy of Distracted Driving Laws in California

**DOI:** 10.3389/fpubh.2019.00207

**Published:** 2019-08-08

**Authors:** Caleb Lyu, Mirna Ponce Jewell, Jennifer Cloud, Lisa V. Smith, Tony Kuo

**Affiliations:** ^1^Children's Medical Services, Los Angeles County Department of Public Health, Los Angeles, CA, United States; ^2^Division of Chronic Disease and Injury Prevention, Los Angeles County Department of Public Health, Los Angeles, CA, United States; ^3^Office of Health Assessment and Epidemiology, Los Angeles County Department of Public Health, Los Angeles, CA, United States; ^4^Department of Epidemiology, University of California, Los Angeles (UCLA), Jonathan and Karin Fielding School of Public Health, Los Angeles, CA, United States; ^5^Department of Family Medicine, David Geffen School of Medicine at UCLA, Los Angeles, CA, United States; ^6^Population Health Program, UCLA Clinical and Translational Science Institute, Los Angeles, CA, United States

**Keywords:** distracted driving, distracted driving laws, low socioeconomic status residents, cellphone use, other distractions

## Abstract

**Objective:** To provide a baseline of various driving behaviors and to identify opportunities for prevention of distracted driving during the infancy of state laws that prohibited cellphone use while operating a motor vehicle, the 2010–2011 Distracted Driving Survey collected information on multiple distracted driving behaviors from lower-income clients of three designated, multi-purpose public health centers in Los Angeles County.

**Methods:** Descriptive and multivariable negative binomial regression analyses were performed to examine patterns of driving distractions using the Distracted Driving Survey dataset (*n* = 1,051).

**Results:** The most common distractions included talking to other passengers (*n* = 912, 86.8%); adjusting the radio, MP3, or cassette player (*n* = 873, 83.1%); and adjusting other car controls (*n* = 838, 79.7%). The median number of distinct distractions per survey participant was 11 (range: 0–32). Factors predicting the number of distinct distractions included being male [incidence rate ratio (IRR): 1.14; 95% confidence interval (CI): 1.06, 1.23], having a lower education (IRR: 0.73; 95% CI: 0.62, 0.84), and having more years of driving experience (IRR: 1.67; 95% CI: 1.33, 2.11). A variety of distractions, including cellphone use and texting, were predictive of increased motor vehicle crashes in the prior 12 months (*p* < 0.05).

**Conclusions:** Distracted driving beyond cellphone use and texting were common in the survey sample, suggesting a need for additional public education and more inclusive distracted driving laws that cover these other activity types.

## Introduction

Distracted driving (DD) is a serious public health problem in the United States (U.S.), resulting in 3,166 and 599 deaths involving drivers and non-occupants, respectively, in 2017 (1). The estimated number of fatalities from distraction-affected crashes has fluctuated very little during the past 5 years−2,923 in 2013 to 2,935 in 2017. This public safety problem is exacerbated by the increasing sources of distraction behind the wheel, in part attributed to the ubiquity of in-vehicle information systems from emergent technologies (2).

In response to this burden of fatality as well as injury, U.S. government entities have begun to implement legislative as well as public education strategies to combat this issue. For example, during the past decade, a number of media campaigns have been launched to educate the public about DD, including the *One Text or Call Could Wreck It All*; *U Drive. U Text. U Pay*.; and GEICO's *Stand Against Distracted Driving*. Additionally, the month of April has now been designated as “Distracted Driving Awareness Month” by the National Safety Council.

While New York became the first state of the union to implement a law prohibiting all drivers from talking on a hand-held cellphone while driving (in 2001), the majority of cellphone and texting laws in other states were passed after 2008 (3). The state of Washington enacted the first law specifically banning all drivers from texting, effective January 1, 2008. Currently nearly all states except Arizona, Montana, and Missouri have texting bans that apply to all drivers (4). However, these cellphone use and texting laws are not uniformly enforced, often varying by state and by whether the statutes called for primary or secondary enforcement.

To date, the effectiveness of U.S. DD laws remains in question (3, 4). For example, distraction-affected crashes can be caused by various activity types other than cellphone use or texting. These non-cellphone or non-texting distractions may include eating or drinking, adjusting music/audio controls, and other interactions with passengers in the same vehicle (5). Little is currently known about these other activities that may represent significant distractions while driving, as few studies have described these distraction behaviors and their frequency within the context of motor vehicle crash risk.

The 2010–2011 Distracted Driving Survey (DDS) was conducted in part to address this gap in public health practice, during a time when DD legislation was still at its infancy (early implementation) in many jurisdictions, and at the federal level. The present study capitalizes on this timing and provides a snapshot of DD (within a historical context) in a sample of low socioeconomic status (SES) residents in Los Angeles County (LAC). The study analyzed the DDS dataset to assess and describe what these distractions were during this pivotal time when the California DD laws were being implemented. The study also examined factors which may have influenced drivers to engage in a variety of distractions while driving.

## Methods

### Study Design and Participants

The 2010–2011 DDS was a cross-sectional survey conducted using a rapid needs assessment approach that answered health assessment questions about driving distractions in a low SES population in LAC. The survey was administered by trained, bilingual Department of Public Health (DPH) staff to all eligible persons attending three designated LAC public health centers during October 4 to December 1, 2010. A systematic, serial sampling protocol was employed on pre-determined dates to screen for eligibility and recruit prospective participants until the first 350 were enrolled at each of the centers. To be eligible, all participants must meet the following criteria: (a) be a client of the health center on the day of recruitment (i.e., receiving services); (b) be at least 16 years of age; (c) be a driver of a motor vehicle; and (d) be able to complete the survey in English or Spanish. All participants provided verbal informed consent prior to enrollment in the survey. Eligible persons under 18 years of age were required to sign an assent form prior to being handed a survey questionnaire; however, the Institutional Review Board that reviewed this study did approve and offered a waiver option. A low-fat granola bar was given as an incentive to all participants who completed the survey. All survey protocols and instruments were reviewed and approved by the DPH Institutional Review Board prior to field implementation (IRB # 2010-09-295).

### Measures and Procedures

The survey was a 4-page self-administered questionnaire developed in English and translated into Spanish by bilingual DPH staff; cultural and linguistic relevance were pretested prior to finalizing the instrument. The survey questionnaire was divided into three parts: (1) questions about socio-demographic characteristics; (2) questions about participants' driving experiences, including recent accident history in the past 12 months; and (3) questions about the distractions that participants engaged in while driving during the past 30 days.

To assess the frequency of engaging in specific distractions, participants were asked: “During the past 30 days, how often did you do this activity while you were driving a car or other vehicle?” Response categories to the question were fielded as: *Never* (0%), *Rarely* (1–10%), *Sometimes* (11–50%), *Most of the time* (51–99%), and *Always* (100%). These variables were dichotomized as “No” if the response was *Never* and “Yes” for all other affirmative responses. “Yes” categories were summed across a single participant to obtain the number of distinct distractions per survey participant, the primary dependent variable of interest.

### Data Analyses

Frequency distributions of key variables were tabulated to inform subsequent analyses. Continuous variables were converted to categorical variables to reflect context and assist with interpretation of results. Age categories were derived from standard ranges used by the Centers for Disease Control and Prevention. Due to sparse data, Native American/Alaskan Native and Mixed/Multiethnic race/ethnicity categories were combined as “Other.” A negative binomial regression model was performed to assess predictors of the number of distinct distractions per survey participant, which is indicated when the dependent variable of interest is depicted as counts and there is overdispersion. Variables included in the model were gender, age, race/ethnicity, education level, health insurance status, years of driving experience, duration of daily non-work-related driving, and duration of one-way travel between home and work. Participants where duration of one-way travel between home and work was not applicable were excluded from the model. Observations with missing data were also excluded from the analyses. The primary measure of interest was the incidence rate ratio, which is the ratio of the mean number of distinct distractions per survey participant for an index group vs. the mean number of distinct distractions for a reference group. In a sub-analysis, binary logistic regression was used to examine the association between specific distractions and involvement in a motor vehicle crash as the driver in the past 12 months. Specifically, potential confounders (age, gender, and race/ethnicity) and explanatory DDS variables (e.g., illicit drug use while driving or reading electronic billboards) were entered simultaneously into the regression models, which generated adjusted odds ratios and 95% confidence intervals (CIs). All analyses were conducted using the SAS 9.4 statistical package (SAS Institute Inc., Cary, North Carolina).

## Results

The DDS was completed by 1,051 participants, for a response rate of 95%. Socio-demographic characteristics and driving experiences of the survey participants are presented in Table 1. Most were 25–34 years of age (*n* = 355, 33.8%), of Hispanic/Latino race/ethnicity (*n* = 451, 42.9%), had at least some sort of college education (*n* = 633, 60.2%), and reported not having health insurance (*n* = 585, 55.7%). For the latter characteristic, the overall population in Los Angeles County, by comparison, contained a much lower percentage of uninsured persons (23.5%). The average number of years of experience driving a motor vehicle was 14.4 (Range: 1–61 years), and the average duration of daily non-work-related driving was 63.3 min (Range: 0–720 min). The average duration of one-way travel between home and work was 25.5 min (Range: 1–120 min) and 10.2% (*n* = 107) reported being involved in a motor vehicle crash in the past 12 months.

**Table 1:** Socio-demographic characteristics and driving experiences of participants from the 2010–2011 Distracted Driving Survey in Los Angeles County.

**Characteristics**	**Distracted driving survey**		**LA county, 2010^b^**		**LA county, 2017^b^**	
	**Total^a^**		**Total**		**Total**	
	***n***	**%**	***n***	**%**	***n***	**%**
All participants	1051	100.0	9,818,605	100.0	10,105,722	100.0
**Gender**						
Female	588	55.9	4,978,951	50.7	5,126,081	50.7
Male	460	43.8	4,839,654	49.3	4,979,641	49.3
**Age**						
>16 (screened for eligibility but age information was not stated in the survey)	2	0.2	–	–	–	–
16 – 24^c^	237	22.5	1,506,418	15.3	1,426,000	14.1
25 – 34	355	33.8	1,475,731	15.0	1,593,895	15.8
35 – 44	252	24.0	1,430,326	14.6	1,397,855	13.8
45 – 54	118	11.2	1,368,947	13.9	1,381,247	13.7
55 and older^c^	87	8.3	1,774,507	18.1	1,874,533	18.5
**Race/Ethnicity^d^**						
Hispanic/Latino	451	42.9	4,687,889	47.7	4,893,579	48.4
African-American/Black	262	24.9	815,086	8.3	799,579	7.9
White/Non-Hispanic	181	17.2	2,728,321	27.8	2,676,982	26.5
Asian/Pacific Islander	109	10.4	1,348,135	13.7	1,467,527	14.5
Other^e^	44	4.2	239,174	2.4	268,055	2.7
**Education level^f^**						
Less than high school	149	14.2	1,540,889	24.2	1,439,724	20.7
High school graduate or GED	245	23.3	1,299,471	20.4	1,446,215	20.8
Some college, community college, or trade school	332	31.6	1,664,604	26.2	1,824,889	26.2
College graduate/postgraduate	301	28.6	1,859,031	29.2	2,244,291	32.2
**Has health insurance^f^**						
Yes	330	31.4	7,464,068	76.5	9,191,163	91.0
No	585	55.7	2,291,120	23.5	905,539	9.0
**Years driving a motor vehicle**						
0 – 9	407	38.7	–	–	–	–
10 – 19	309	29.4	–	–	–	–
20 – 29	182	17.3	–	–	–	–
30 and over	132	12.6	–	–	–	–
**Duration of daily non-work-related driving (Minutes)**						
0 – 30	415	39.5	–	–	–	–
31 – 60	327	31.1	–	–	–	–
>60	227	21.6	–	–	–	–
**Duration of one-way travel between home and work (Minutes)^f^**						
1 – 14	121	11.5	815,880	20.1	760,289	16.6
15 – 19	110	10.5	577,279	14.2	619,919	13.5
20 – 29	161	15.3	811,044	19.9	843,912	18.4
30 and greater	250	23.8	1,864,047	45.8	2,361,607	51.5
Not applicable	357	34.0	–	–	–	–
**Involved in motor vehicle crash in past 12 Months**						
Yes	107	10.2	–	–	–	–
No	890	84.7	–	–	–	–

a *Excluded missing values; total percentages may exceed 100% due to rounding*.

b *Sources: U.S. Census Bureau, 2010 Census, 2010 American Community Survey, and 2017 American Community Survey*.

c *For LA County population, the age range is 15–24 and 55–79 years*.

d *For LA County population, the race categories are Hispanic/Latino, Non-Hispanic Black/African American, Non-Hispanic White, Non-Hispanic Asian/Pacific Islander, and Non-Hispanic Other race/ethnicity*.

e *Other race/ethnicity included Native American/Alaskan Native and Mixed/Multiethnic*.

f *For LA County population, the education level is for population 25 years and older, health insurance of civilian non-institutionalized population, and travel time to work for workers 16 years and over who did not work at home*.

The median number of distinct distraction activities that participants engaged in during the past 30 days was 11 and ranged from 0 to 32 (skewness = 0.29; kurtosis = 0.13). The most common distraction was talking to other passengers (*n* = 912, 86.8%) (Table 2). Other common distractions included adjusting the radio, MP3, or cassette player (*n* = 873, 83.1%); adjusting the controls in the car including air conditioner, sunroof, mirrors, etc. (*n* = 838, 79.7%); eating or drinking (*n* = 817, 77.7%); and singing (*n* = 794, 75.5%).

**Table 2:** Self-reported distractions in the past 30 days, 2010–2011 Distracted Driving Survey in Los Angeles County.

**Driving distraction**	**Frequency**											
	**Total^a^**		**Always**		**Most of the time**		**Sometimes**		**Rarely**		**Never**	
	***n***	**%**	***n***	**%**	***n***	**%**	***n***	**%**	***n***	**%**	***n***	**%**
Talked to other passengers	912	86.8	160	15.2	272	25.9	355	33.8	125	11.9	120	11.4
Adjusted the radio, MP3, cassette player	873	83.1	169	16.1	233	22.2	322	30.6	149	14.2	166	15.8
Adjusted the controls in car including air conditioner, sunroof, mirrors, etc.	838	79.7	102	9.7	138	13.1	369	35.1	229	21.8	199	18.9
Ate food or drank beverages	817	77.7	49	4.7	88	8.4	438	41.7	242	23.0	217	20.6
Sang a song	794	75.5	100	9.5	155	14.7	334	31.8	205	19.5	244	23.2
Read street billboards	767	73.0	44	4.2	69	6.6	370	35.2	284	27.0	258	24.5
Read electronic billboards	692	65.8	39	3.7	56	5.3	323	30.7	274	26.1	311	29.6
Watched a car crash that already occurred	643	61.2	28	2.7	30	2.9	202	19.2	383	36.4	387	36.8
Talked on a cellphone with a legal hands-free device	582	55.4	73	6.9	112	10.7	247	23.5	150	14.3	449	42.7
Sent or received text messages	526	50.0	35	3.3	65	6.2	200	19.0	226	21.5	503	47.9
Used GPS or navigation system	450	42.8	46	4.4	81	7.7	187	17.8	136	12.9	579	55.1
Kissed, cuddled, or held passenger's hand	422	40.2	22	2.1	37	3.5	162	15.4	201	19.1	613	58.3
Tended to children in car	415	39.5	28	2.7	47	4.5	158	15.0	182	17.3	621	59.1
Daydreamed	381	36.3	12	1.1	21	2.0	109	10.4	239	22.7	657	62.5
Got dressed or undressed	319	30.4	6	0.6	7	0.7	86	8.2	220	20.9	721	68.6
Sent or received emails	287	27.3	25	2.4	20	1.9	89	8.5	153	14.6	741	70.5
Tended to personal hygiene	273	26.0	24	2.3	20	1.9	65	6.2	164	15.6	753	71.6
Danced	272	25.9	30	2.9	17	1.6	112	10.7	113	10.8	761	72.4
Smoked cigarettes or cigars	230	21.9	42	4.0	40	3.8	85	8.1	63	6.0	808	76.9
Meditated	201	19.1	14	1.3	16	1.5	79	7.5	92	8.8	826	78.6
Read or wrote	176	16.7	5	0.5	6	0.6	52	4.9	113	10.8	861	81.9
Drove under the influence of alcohol	178	16.9	6	0.6	5	0.5	38	3.6	129	12.3	862	82.0
Physically hit or slapped someone riding in car	140	13.3	15	1.4	3	0.3	39	3.7	83	7.9	897	85.3
Fell asleep	135	12.8	2	0.2	4	0.4	22	2.1	107	10.2	905	86.1
Engaged in sexual intercourse or activity	118	11.2	10	1.0	2	0.2	35	3.3	71	6.8	920	87.5
Did paperwork for work or homework for school	107	10.2	9	0.9	7	0.7	33	3.1	58	5.5	930	88.5
Used illicit drugs	93	8.8	6	0.6	12	1.1	35	3.3	40	3.8	945	89.9
Drove under the influence of prescription drugs that may impair driving	91	8.7	6	0.6	5	0.5	23	2.2	57	5.4	949	90.3
Held children or pets in lap	89	8.5	5	0.5	6	0.6	22	2.1	56	5.3	951	90.5
Used a laptop computer	67	6.4	11	1.0	7	0.7	18	1.7	31	2.9	963	91.6
Played games on cellphone or electronic gaming system	59	5.6	13	1.2	6	0.6	15	1.4	25	2.4	974	92.7
Watched television or videos	55	5.2	7	0.7	0	0.0	19	1.8	29	2.8	982	93.4

a *Total number of participants engaging in the activity at least rarely, sometimes, most of the time, and always*.

There were several characteristics of the study sample that predicted the number of distinct distractions per survey participant. Male participants, for example, were more likely to have a higher number of distinct distractions [incidence rate ratio (IRR) = 1.14, 95% confidence interval (CI) = 1.06, 1.23], as were those who had a higher number of years of driving experience (e.g., 30 years and over: IRR = 1.67, 95% CI = 1.33, 2.11) (Table 3). Those in older age categories were less likely to have engaged in distinct distractions, with the 55 and older group being the least likely to engage in a variety of distractions (IRR = 0.39, 95% CI = 0.30, 0.51). Those who had completed less than high school education (IRR = 0.73, 95% CI = 0.62, 0.84), high school or GED level of education (IRR = 0.72, 95% CI = 0.64, 0.80), and some college, community college, or trade school (IRR = 0.88, 95% CI = 0.80, 0.96) were also less likely to engage in a variety of distractions, as compared to those with college graduate/postgraduate education.

**Table 3:** Predictors of the number of distinct distractions while driving per survey participant in the past 30 days, 2010–2011 Distracted Driving Survey in Los Angeles County.

**Characteristic (reference)**	**IRR^a^**	**95% CI^a^**	***p*-value^a^**
**Gender (Referent = Female)**			
Male	1.14	1.06, 1.23	<0.01
**Age (Referent = 16 – 24)**			
25 – 34	0.84	0.74, 0.94	<0.01
35 – 44	0.58	0.50, 0.68	<0.01
45 – 54	0.49	0.40, 0.60	<0.01
55 and older	0.39	0.30, 0.51	<0.01
**Race (Referent = White/Non-Hispanic)**			
African-American/Black	1.05	0.93, 1.17	0.45
Asian/Pacific Islander	1.00	0.87, 1.15	0.98
Hispanic/Latino	0.88	0.78, 0.98	0.02
Other	1.07	0.88, 1.30	0.51
**Education (Referent = College Graduate/Postgraduate)**			
Completed less than high school	0.73	0.62, 0.84	<0.01
High school graduate or GED	0.72	0.64, 0.80	<0.01
Some college, community college, or trade school	0.88	0.80, 0.96	0.01
**Years of driving experience (Referent = 0 – 9)**			
10 – 19	1.16	1.03, 1.29	0.01
20 – 29	1.42	1.20, 1.68	<0.01
30 and over	1.67	1.33, 2.11	<0.01

*CI, confidence interval; IRR, incident rate ratio*.

a *Estimates obtained from the simultaneous entry of all covariates in the table, having health insurance (yes/no), duration of daily non-work-related driving (0–30, 31–60, >60 min), and duration of one-way travel between home and work (1–15, 16–20, 21–30, >30 min) into a negative binomial regression model*.

Although not the primary focus of the present study, a sub-analysis of the DDS found that there were many activities other than cellphone use or texting that were associated with being involved in a motor vehicle crash in the past 12 months (Table 4). Results from the binary logistic regression suggest that both common (highly prevalent) and rare activities were dangerous to participate in while driving. For example, only 8.8% of the participants used illicit drugs while driving but had 2.87 times the odds (adjusted) of being involved in a crash in the prior 12 months than those who did not (95% CI = 1.61, 5.12). In contrast, 65.8% of all participants reported reading electronic billboards. These participants had 1.82 times the odds (adjusted) of being involved in a crash as compared to those who did not (95% CI = 1.07, 3.11). Other activities with significant results (*p* < 0.05) in this analysis included physically hitting someone riding in the car, engaging in sexual intercourse, tending to personal hygiene, dancing, playing games on cell phone or other gaming device, using a handsfree device, watching a prior car crash, falling asleep, driving under the influence of prescription drugs that may impair driving, daydreaming, and driving under the influence of alcohol. Lastly, cellphone use while driving did not have the highest odds of being in a motor vehicle accident in the prior 12 months.

**Table 4:** Associations between self-reported distracted driving activities in the past 30 days and being involved in a car/motor vehicle crash in the past 12 months (*n* = 1,051); 2010–2011 Distracted Driving Survey in Los Angeles County.

**Activity**	**Total^a^**		**Association^b^**	
	***n***	**%**	**aOR**	**95% CI**
**Driving distractions**				
Physically hit or slapped someone riding in car	140	13.3	3.22	1.95, 5.30^***^
Engaged in sexual intercourse or activity	118	11.2	2.99	1.71, 5.22^***^
Used illicit drugs	93	8.8	2.87	1.61, 5.12^***^
Tended to personal hygiene	273	26.0	2.25	1.47, 3.45^***^
Danced	272	25.9	2.11	1.35, 3.30^**^
Played games on cell phone or electronic gaming system	59	5.6	2.07	1.02, 4.21^*^
Watched television or videos	55	5.2	2.02	0.95, 4.29
Ate food or drank beverages	817	77.7	1.90	0.98, 3.68
Read electronic billboards	692	65.8	1.82	1.07, 3.11^*^
Watched a car crash that already occurred	643	61.2	1.69	1.06, 2.69^*^
Talked on cell phone with a legal hands-free device	582	55.4	1.65	1.05, 2.60^*^
Smoked cigarettes or cigars	230	21.9	1.60	1.00, 2.56^*^
Used a laptop computer	67	6.4	1.60	0.77, 3.31
Sent or received text messages	526	50.0	1.57	0.98, 2.54
Sent or received emails	287	27.3	1.52	0.97, 2.38
Got dressed or undressed	319	30.4	1.48	0.95, 2.30
Adjusted the controls in car including air conditioning, sunroof, mirrors, etc.	838	79.7	1.47	0.79, 2.76
Held children or pets in lap	89	8.5	1.40	0.74, 2.65
Kissed, cuddled, or held passenger's hand	422	40.2	1.36	0.89, 2.08
Read or wrote	176	16.7	1.35	0.81, 2.24
Used GPS or navigation system	450	42.8	1.28	0.83, 1.97
Read street billboards	767	73.0	1.18	0.70, 2.00
Talked to other passengers	912	86.8	1.18	0.57, 2.46
Did paperwork for work or homework for school	107	10.2	1.15	0.61, 2.16
Adjusted the radio, MP3, or cassette player	873	83.1	0.97	0.51, 1.85
Tended to children in car	415	39.5	0.91	0.59, 1.39
Sang a song	794	75.5	0.80	0.48, 1.34
**Driving impairments**				
Fell asleep	135	12.8	2.71	1.62, 4.55^***^
Drove under the influence of prescription drugs that may impair driving	91	8.7	2.68	1.50, 4.78^**^
Daydreamed	381	36.3	2.00	1.31, 3.07^**^
Drove under the influence of alcohol	178	16.9	1.87	1.15, 3.04^*^
Meditated	201	19.1	1.11	0.67, 1.84

*CI, confidence interval*.

* *p < 0.05*,

** p < 0.01, and

*** *p < 0.001*.

a Total number of survey participants engaging in the activity at least “Rarely,” “Sometimes,” “Most of the time,” and “Always.”

b *Adjusted odds ratio (aOR) values were generated by simultaneous entry of covariates into a logistic regression model, controlling for Age (16-24, 25-34, 35-44, 45-54, 55-78) Race (African American/Black, Asian/Pacific Islander, Hispanic/Latino, White/Non-Hispanic, Other); and Gender (Male, Female)*.

## Discussion

The DDS found that gender, age, education level, and years of driving experience predicted the number of distinct distractions among survey participants. Gender and age, in particular, are supported by prior research which has documented their association to DD (6). Likewise, higher education (e.g., Bachelor's degree or greater) has been shown by Hoff and colleagues to predict greater variety of DD behaviors (7); this is similar to what the DDS has reported for participants with a college education. Interestingly, among survey participants with a longer driving experience, the variety and frequency of driving distractions were higher than for those with a shorter driving experience. Although somewhat contradictory to McEvoy and co-workers' finding, which showed that drivers with a shorter driving experience were more likely to be engaged in DD at the time of a crash (8), the present result on this subject matter was not necessarily inconsistent with what is known about driving skills, as more experienced drivers may also be more adept at avoiding motor vehicle crashes despite engaging in more DD activities.

In general, driving distractions have been found to be associated with motor vehicle crashes. In a study analyzing the 100-Car Naturalistic Driving Study data, behaviors such as engaging in moderate and complex secondary tasks while driving, including looking at external objects, reading, and applying makeup, resulted in at least two to three times the odds of being involved in a near-crash/crash event (9). In the DDS sub-analysis, similar relationships between DD behaviors and crashes were observed.

Because the danger of DD can be debilitating and/or fatal, there is a need for additional public education and more inclusive DD laws that consider other distraction types beyond just cellphone use or texting. Existing DD legislation, for example, could be further refined to cover the total number and types of distractions that drivers may engage in. Only a few states - Connecticut, Maine, Oklahoma, and Utah - and the District of Columbia currently have language in their driving laws to address other forms of DD. Past policy efforts, such as seat belt laws and alcohol-impaired driving regulations, suggest that combining public education with strong regulation and enforcement of the laws can be quite effective for promoting safer driving conditions and for reducing crash deaths over time (10, 11).

Although the DDS was conducted in 2010, the survey offers an opportunity to provide a snapshot of driving behaviors among a lower SES population during the early stages of DD law implementation in California; the bans for cellphone use and texting were instituted in 2008 and 2009, respectively (California Vehicle Code, 2008. §23123; California Vehicle Code, 2009. §23123.5). Recent literature suggests that efforts to curb DD through texting bans and to reduce its negative consequences were associated with significant decreases in the incidence of emergency department visits that follow a collision (12).

The DDS provides added value to public health practice because its scope (i.e., asked about a multitude of driving distractions) is broader than most studies in the literature. That is, baseline information from the study can be used to design roadway (streetscape modifications) and driver interventions (public education and more inclusive DD laws) for decreasing motor vehicle-related fatalities and injuries in LAC. Such data, for instance, could be applied to aid program development in the *Vision Zero* initiative. This initiative's primary goal is to eliminate fatalities from motor vehicle-pedestrian/-bicyclist collisions, from present level to zero by 2035 (13).

The DDS also adds value because the data that it generated have immediate application in the public health center setting. Since the survey findings were about their clients, physicians and public health nurses are likely to be more motivated to counsel on this personal safety issue, much like they do for seat belt use during a clinic visit.

### Limitations

In spite of its unique strengths (e.g., relevance to local public health practice and injury prevention), the DDS has several limitations. First, the data is almost 8 years old—i.e., study findings may not reflect current attitudes or behaviors. Nonetheless, the DDS data do offer a historical reference which can be used as comparison for impact evaluation of DD education campaigns and legislation and future research efforts on this topic. Second, the DDS sample may not be representative of the general population, but rather of the lower SES group that frequent public health centers. However, despite this limitation, findings from another study suggest that the prevalence of DD may be even greater among populations with higher SES, reflecting a greater need for intervention in these groups (14). There are also mixed results on income levels and its relationship to motor vehicle casualties in the literature, with some showing a higher fatality rate among those in lower income areas while others showing no association (15, 16). Third (and lastly), since the survey relied on self-reported data, a number of biases could have altered the interpretation of the results, including but not limited to recall and social desirability biases.

## Conclusions

The present analysis of the DDS provides a unique narrative of a multitude of driving behaviors during the early implementation of DD legislation in California. It also provides a sample reference that future research can compare to. The survey findings highlight the heterogeneity of driving distractions beyond just cellphone use and texting—many individuals were talking, eating, grooming, and adjusting various car features while driving, to name a few of these activities. Differences in SES aside, DD is a preventable phenomenon that can affect more than the person participating in the activity. As such, it is a worthwhile public health intervention priority. Taken together with other literature, the DDS data suggest that revisiting current DD laws to broaden their scopes and educating the public through more robust media or public service announcement campaigns, are both worthy investments, and likely actions that should be taken to address this public health problem. The sheer variety of local DD activities as described by the DDS—a survey conducted during a time when DD laws were becoming more commonplace—have policy implications and practical applications for those interested in reducing DD morbidity and mortality in their jurisdictions, both in the U.S. and abroad.

## Data Availability

Access to the datasets are available on request to the corresponding author and will be considered on a case by case basis.

## Ethics Statement

All survey protocols and instruments were reviewed and approved by the DPH Institutional Review Board prior to field implementation (IRB # 2010-09-295). This is stated in the manuscript as well.

## Author Contributions

CL led the analysis and writing of this article. LS and TK conceptualized the original study design. JC and MP supervised the data collection and management. All authors participated in the interpretation of the results, edited the manuscript for intellectual content and accuracy, and reviewed and approved this submission.

### Conflict of Interest Statement

The authors declare that the research was conducted in the absence of any commercial or financial relationships that could be construed as a potential conflict of interest.
